# Efficacy of sifalimumab for treatment of skin injury caused by systemic lupus erythematosus

**DOI:** 10.1097/MD.0000000000017607

**Published:** 2019-10-25

**Authors:** Ai-xin Huo, Wen-hui Chen, Yu-hong Liu, Peng Gao, Jing Li

**Affiliations:** a Department of Immunology and Rheumatology, Yanan University Affiliated Hospital, Yan’an; b Department of Dermatology, Shaanxi Provincial Institute of Dermatology and Venereology, Xi’an, China.

**Keywords:** efficacy, safety, sifalimumab, skin injury, systemic lupus erythematosus

## Abstract

**Background:**

This study aims to provide the best possible evidence-based information on the efficacy and safety of sifalimumab for treatment of skin injury (SI) caused by systemic lupus erythematosus (SLE).

**Methods:**

In this study, electronic databases of MEDLINE, EMBASE, Cochrane Library, PsycINFO, CINAHL Plus, Global Health, WHO Global Index Medicus, Virtual Health Library, Social Care Online, Cumulative Index to Nursing and Allied Health Literature, Allied and Complementary Medicine Database, Chinese Biomedical Literature Database, and China National Knowledge Infrastructure will be searched comprehensively from inceptions to June 30, 2019 without language restrictions. We will include randomized controlled trials (RCTs) on evaluating the efficacy and safety of sifalimumab for SI caused by SLE. Two investigators will conduct study selection, data extraction, and risk of bias assessment independently. We will use RevMan 5.3 Software to perform statistical analysis.

**Results:**

This study will lie in the exhaustive and systematic nature of the literature search and its methods for evaluating quality and analyzing RCTs data. Considering the controversial efficacy of the treatment for sifalimumab, this study is responsible for improving the existing evidence on the efficacy and safety of sifalimumab for SI caused by SLE.

**Conclusion:**

The results of this study will provide latest evidence for judging whether sifalimumab is an effective intervention for patients with SI caused by SLE or not.

**Study registration:**

CRD42019148225.

## 1 Introduction

Systemic lupus erythematosus (SLE) is a serious chronic autoimmune disease,^[1–3]^ which characterized by a wide spectrum of clinical and serological symptoms.^[4–6]^ It mainly manifests as joint pain and swelling, chest pain, fever, general discomfort, hair loss, weight loss, mouth sores, sensitivity to sunlight and skin rash, swollen lymph nodes, and skin injury (SI) in some patients.^[6–10]^ Previous studies have found that several factors may be responsible for this disorder, such as genetic, environmental, hormonal, and certain medicines.^[11–16]^ It has been estimated that its prevalence and incidence are about 100–150/100,000 persons and more than 5/100,000 people annually, respectively.^[17–19]^ Although a variety of managements are reported to treat SI caused by SLE, their efficacy is still limited.^[20–24]^ Fortunately, sifalimumab is reported to treat patients with SI caused by SLE.^[25–29]^ However, its results are still inconsistent. Therefore, this study will systematically assess the efficacy and safety for the treatment of patients with SI caused by SLE.

## 2 Methods and analysis

### 2.1 Ethics and dissemination

This study is secondary analysis of published studies; therefore, no ethical approval is needed. Planned disseminations include a peer-reviewed publication and conference proceedings.

### 2.2 Inclusion criteria for study selection

#### 2.2.1 Types of studies

We will include all published and unpublished randomized controlled trials (RCTs), comparing sifalimumab with other treatments for patients with SI caused by SLE. All other studies except RCTs will be excluded.

#### 2.2.2 Types of participants

Participants with a clinically confirmed diagnosis of SI caused by SLE will be considered for inclusion regardless their race, gender, age, education, or economic status.

#### 2.2.3 Types of interventions

Any forms of sifalimumab in the experimental group will be included.

Any interventions, except sifalimumab in the control group will be considered for inclusion.

#### 2.2.4 Type of outcome measurements

Primary outcomes include time to complete healing of injury skin, and number of SI healed.

Secondary outcomes consist of hospital readmission rate, SLE Response Index, SLE Flare Index rate, changes in inflammatory and hemostatic markers, and adverse events.

### 2.3 Literature search

We will comprehensively carry out searches in bibliographic databases of MEDLINE, EMBASE, Cochrane Library, PsycINFO, CINAHL Plus, Global Health, WHO Global Index Medicus, Virtual Health Library, Social Care Online, Cumulative Index to Nursing and Allied Health Literature, Allied and Complementary Medicine Database, Chinese Biomedical Literature Database, and China National Knowledge Infrastructure. We will search all databases from inceptions to June 30, 2019 without language restrictions. Exemplary search strategy for MEDLINE is provided in Table 1. We will apply other similar search strategies to other electronic databases. Additionally, we will also search unpublished and conference proceedings to avoid any missing potential studies.

**Table 1:**
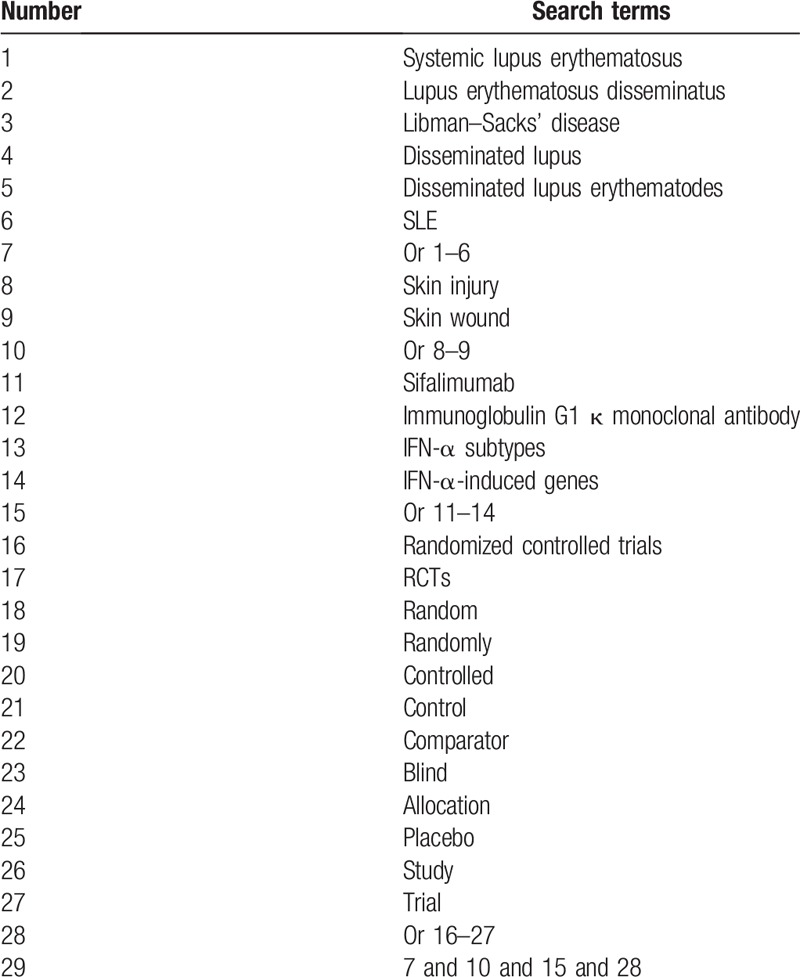
Search strategy of MEDLINE database.

### 2.4 Data collection and management

#### 2.4.1 Study selection

For studies obtained via all literature records, 2 investigators will independently scan titles and abstracts of all studies and retrieve potentially relevant studies. After that, they will also review full-texts against all inclusion criteria. Any disagreements between 2 authors will be solved by consensus with a 3rd independent investigator. The process of study selection will be presented in the flowchart.

#### 2.4.2 Data extraction and management

A data collection sheet will be designed before data extraction. Two investigators will independently extract relevant details about the study design, study methods, and outcome results. Any divergences will be solved by consensus or by independent assessment by a 3rd investigator. The extracted information will consist of title, study year and author, study region and setting, study design, sample size, eligibility criteria, baseline characteristics, intervention details, comparisons, treatment details, study methods, outcome measurements, safety, and funding resources.

#### 2.4.3 Dealing with missing data

When information regarding any of the above is unclear or insufficient, we will contact primary author of the original studies in order to ask for further details. We will pool the available data if further details cannot be getable.

### 2.5 Assessment of risk of bias in included studies

Two independent investigators will use Cochrane Collaboration's “Risk of bias” tool for included RCTs and eligibility criteria in the Cochrane Handbook for Systematic Reviews of Interventions to assess those in the associated domains of the reported methods and outcome results. Any disagreements between 2 independent investigators will be solved by a 3rd investigator through discussion.

### 2.6 Measures of treatment effect

#### 2.6.1 Dichotomous data

For dichotomous data, we will exert the results as risk ratio with 95% confidence intervals.

#### 2.6.2 Continuous data

For continuous data, we will utilize the results as mean difference or standardized mean difference with 95% confidence intervals.

### 2.7 Assessment of heterogeneity

We will evaluate statistical heterogeneity using *I*^2^ statistic by 2 independent investigators. We will consider heterogeneity as acceptable if *I*^2^ is 50% or less, and a fixed-effects model will be used. We will consider heterogeneity as substantial if *I*^2^ is more than 50%, and a random-effects model will be applied.

### 2.8 Assessment of reporting biases

We will apply funnel plots and Eggers Regression test^[30,31]^ to assess publication bias when at least 10 RCTs are available for meta-analysis.

### 2.9 Data analysis

We will apply RevMan 5.3 software for data analysis. If heterogeneity is acceptable among included studies (*I*^2^ ≤ 50%), we will carry out meta-analysis when it is possible. If heterogeneity is substantial among included studies (*I*^2^ > 50%), we will perform subgroup analysis. If there is still significant heterogeneity after subgroup analysis, we will not pool the data, and report outcome results as a narrative review.

### 2.10 Subgroup analysis

Subgroup analysis will be exerted according to the different treatments, comparators, and outcome measurements to explore any possible reasons that may cause such significant heterogeneity.

### 2.11 Sensitivity analysis

We will conduct a sensitivity analysis to check robustness of outcome results by excluding studies with high risk of bias.

## 3 Discussion

SLE is a chronic, autoimmune, inflammatory disorder that often involves several systems and organs in patients with such condition. Some of such patients also have SI. Previous studies have highlighted the role of sifalimumab for the treatment of patients with SI caused by SLE. However, the conclusion is still inconsistent. This study aims to systematically investigate the efficacy and safety of sifalimumab for SI secondary to SLE.

This study will comprehensively and systematically search more potential literatures to find more eligible high quality studies. It may present solid data and robust evidence, as well as provide helpful recommendation for both patients and clinical practice.

## Acknowledgments

The authors thank Yan’an Specialized Project for Transformation and Promotion of Achievements (2018CGZH-15) for the support. The funder had no role in this study.

## Author contributions

**Conceptualization:** Ai-xin Huo, Wen-hui Chen, Yu-hong Liu, Peng Gao, Jing Li.

**Data curation:** Ai-xin Huo, Wen-hui Chen, Jing Li.

**Formal analysis:** Ai-xin Huo, Yu-hong Liu, Peng Gao.

**Investigation:** Wen-hui Chen.

**Methodology:** Ai-xin Huo, Wen-hui Chen, Yu-hong Liu, Peng Gao, Jing Li.

**Project administration:** Wen-hui Chen.

**Resources:** Ai-xin Huo, Yu-hong Liu, Peng Gao, Jing Li.

**Software:** Ai-xin Huo, Yu-hong Liu, Peng Gao, Jing Li.

**Supervision:** Wen-hui Chen.

**Validation:** Ai-xin Huo, Wen-hui Chen, Peng Gao, Jing Li.

**Visualization:** Ai-xin Huo, Wen-hui Chen, Yu-hong Liu, Jing Li.

**Writing – original draft:** Ai-xin Huo, Wen-hui Chen, Yu-hong Liu, Peng Gao, Jing Li.

**Writing – review & editing:** Ai-xin Huo, Wen-hui Chen, Yu-hong Liu, Peng Gao, Jing Li.

## References

[1] Zucchi D Elefante E Calabresi E One year in review 2019: systemic lupus erythematosus. Clin Exp Rheumatol 2019;37:715 22. 31376267

[2] Dörner T Furie R Novel paradigms in systemic lupus erythematosus. Lancet 2019;393:2344 58. 31180031 10.1016/S0140-6736(19)30546-X

[3] Durcan L O’Dwyer T Petri M Management strategies and future directions for systemic lupus erythematosus in adults. Lancet 2019;393:2332 43. 31180030 10.1016/S0140-6736(19)30237-5

[4] Fava A Petri M Systemic lupus erythematosus: diagnosis and clinical management. J Autoimmun 2019;96:1 3. 30448290 10.1016/j.jaut.2018.11.001 PMC6310637

[5] Kokosi M Lams B Agarwal S Systemic lupus erythematosus and antiphospholipid antibody syndrome. Clin Chest Med 2019;40:519 29. 31376888 10.1016/j.ccm.2019.06.001

[6] Li Q Wu H Liao W A comprehensive review of immune-mediated dermatopathology in systemic lupus erythematosus. J Autoimmun 2018;93:1 5. 30017673 10.1016/j.jaut.2018.07.007

[7] Bortoluzzi A Silvagni E Furini F Peripheral nervous system involvement in systemic lupus erythematosus: a review of the evidence. Clin Exp Rheumatol 2019;37:146 55. 29846158

[8] Olesińska M Saletra A Quality of life in systemic lupus erythematosus and its measurement. Reumatologia 2018;56:45 54. 29686443 10.5114/reum.2018.74750 PMC5911658

[9] Jafri K Patterson SL Lanata C Central nervous system manifestations of systemic lupus erythematosus. Rheum Dis Clin North Am 2017;43:531 45. 29061240 10.1016/j.rdc.2017.06.003

[10] Golder V Hoi A Systemic lupus erythematosus: an update. Med J Aust 2017;206:215 20. 28301792 10.5694/mja16.01229

[11] Pan Q Chen J Guo L Mechanistic insights into environmental and genetic risk factors for systemic lupus erythematosus. Am J Transl Res 2019;11:1241 54. 30972159 PMC6456562

[12] Gergianaki I Bortoluzzi A Bertsias G Update on the epidemiology, risk factors, and disease outcomes of systemic lupus erythematosus. Best Pract Res Clin Rheumatol 2018;32:188 205. 30527426 10.1016/j.berh.2018.09.004

[13] Nevskaya T Gamble MP Pope JE A meta-analysis of avascular necrosis in systemic lupus erythematosus: prevalence and risk factors. Clin Exp Rheumatol 2017;35:700 10. 28240590

[14] Teruel M Alarcón-Riquelme ME The genetic basis of systemic lupus erythematosus: what are the risk factors and what have we learned. J Autoimmun 2016;74:161 75. 27522116 10.1016/j.jaut.2016.08.001

[15] Benvenuti F Gatto M Larosa M Cardiovascular risk factors, burden of disease and preventive strategies in patients with systemic lupus erythematosus: a literature review. Expert Opin Drug Saf 2015;14:1373 85. 26212119 10.1517/14740338.2015.1073259

[16] Ntatsaki E Isenberg D Risk factors for renal disease in systemic lupus erythematosus and their clinical implications. Expert Rev Clin Immunol 2015;11:837 48. 25973642 10.1586/1744666X.2015.1045418

[17] Chakravarty EF Bush TM Manzi S Prevalence of adult systemic lupus erythematosus in California and Pennsylvania in 2000: estimates obtained using hospitalization data. Arthritis Rheum 2007;56:2092 4. 17530651 10.1002/art.22641 PMC2530907

[18] Feldman CH Hiraki LT Liu J Epidemiology and sociodemographics of systemic lupus erythematosus and lupus nephritis among US adults with Medicaid coverage, 2000–2004. Arthritis Rheum 2013;65:753 63. 23203603 10.1002/art.37795 PMC3733212

[19] Furst DE Clarke AE Fernandes AW Incidence and prevalence of adult systemic lupus erythematosus in a large US managed-care population. Lupus 2013;22:99 105. 23042822 10.1177/0961203312463110

[20] Lima GL Paupitz JA Aikawa NE A randomized double-blind placebo-controlled trial of vitamin D supplementation in juvenile-onset systemic lupus erythematosus: positive effect on trabecular microarchitecture using HR-pQCT. Osteoporos Int 2018;29:587 94. 29152675 10.1007/s00198-017-4316-5

[21] Lai ZW Kelly R Winans T Sirolimus in patients with clinically active systemic lupus erythematosus resistant to, or intolerant of, conventional medications: a single-arm, open-label, phase 1/2 trial. Lancet 2018;391:1186 96. 29551338 10.1016/S0140-6736(18)30485-9 PMC5891154

[22] Merrill JT Shanahan WR Scheinberg M Phase III trial results with blisibimod, a selective inhibitor of B-cell activating factor, in subjects with systemic lupus erythematosus (SLE): results from a randomised, double-blind, placebo-controlled trial. Ann Rheum Dis 2018;77:883 9. 29563108 10.1136/annrheumdis-2018-213032

[23] Clowse ME Wallace DJ Furie RA Efficacy and safety of epratuzumab in moderately to severely active systemic lupus erythematosus: results from two phase III randomized, double-blind, placebo-controlled trials. Arthritis Rheumatol 2017;69:362 75. 27598855 10.1002/art.39856 PMC5299488

[24] Boström C Elfving B Dupré B Effects of a one-year physical activity programme for women with systemic lupus erythematosus – a randomized controlled study. Lupus 2016;25:602 16. 26768748 10.1177/0961203315622817

[25] Khamashta M Merrill JT Werth VP Sifalimumab, an anti-interferon-(monoclonal antibody, in moderate to severe systemic lupus erythematosus: a randomised, double-blind, placebo-controlled study. Ann Rheum Dis 2016;75:1909 16. 27009916 10.1136/annrheumdis-2015-208562 PMC5099191

[26] Zheng B Yu XQ Greth W Population pharmacokinetic analysis of sifalimumab from a clinical phase IIb trial in systemic lupus erythematosus patients. Br J Clin Pharmacol 2016;81:918 28. 26659791 10.1111/bcp.12864 PMC4834601

[27] Narwal R Roskos LK Robbie GJ Population pharmacokinetics of sifalimumab, an investigational anti-interferon-(monoclonal antibody, in systemic lupus erythematosus. Clin Pharmacokinet 2013;52:1017 27. 23754736 10.1007/s40262-013-0085-2 PMC3824374

[28] Petri M Wallace DJ Spindler A Sifalimumab, a human anti-interferon-(monoclonal antibody, in systemic lupus erythematosus: a phase I randomized, controlled, dose-escalation study. Arthritis Rheum 2013;65:1011 21. 23400715 10.1002/art.37824 PMC3654174

[29] Merrill JT Wallace DJ Petri M Safety profile and clinical activity of sifalimumab, a fully human anti-interferon (monoclonal antibody, in systemic lupus erythematosus: a phase I, multicentre, double-blind randomised study. Ann Rheum Dis 2011;70:1905 13. 21798883 10.1136/ard.2010.144485

[30] Higgins JPT Green S Cochrane Handbook for Systematic Reviews of Interventions Version 5.1.0. The Cochrane Collaboration, 2011. Available at: http://www.cochrane-handbook.org (Accessed Mar, 2011)

[31] Egger M Davey Smith G Schneider M Bias in meta-analysis detected by a simple, graphical test. BMJ 1997;315:629 34. 9310563 10.1136/bmj.315.7109.629 PMC2127453

